# Regeneration of Articular Cartilage in Lizard Knee from Resident Stem/Progenitor Cells

**DOI:** 10.3390/ijms160920731

**Published:** 2015-09-01

**Authors:** Lorenzo Alibardi

**Affiliations:** Comparative Histolab and Department of Bigea, University of Bologna, via Selmi 3, 40126 Bologna, Italy; E-Mail: lorenzo.alibardi@unibo.it; Tel.: +39-051-209-4142

**Keywords:** lizard, knee, cartilage regeneration, immunocytochemistry, resident stem cells

## Abstract

The epiphysis of femur and tibia in the lizard *Podarcis muralis* can extensively regenerate after injury. The process involves the articular cartilage and metaphyseal (growth) plate after damage. The secondary ossification center present between the articular cartilage and the growth plate is replaced by cartilaginous epiphyses after about one month of regeneration at high temperature. The present study analyzes the origin of the chondrogenic cells from putative stem cells located in the growing centers of the epiphyses. The study is carried out using immunocytochemistry for the detection of 5BrdU-labeled long retaining cells and for the localization of telomerase, an enzyme that indicates stemness. The observations show that putative stem cells retaining 5BrdU and positive for telomerase are present in the superficial articular cartilage and metaphyseal growth plate located in the epiphyses. This observation suggests that these areas represent stem cell niches lasting for most of the lifetime of lizards. In healthy long bones of adult lizards, the addition of new chondrocytes from the stem cells population in the articular cartilage and the metaphyseal growth plate likely allows for slow, continuous longitudinal growth. When the knee is injured in the adult lizard, new populations of chondrocytes actively producing chondroitin sulfate proteoglycan are derived from these stem cells to allow for the formation of completely new cartilaginous epiphyses, possibly anticipating the re-formation of secondary centers in later stages. The study suggests that in this lizard species, the regenerative ability of the epiphyses is a pre-adaptation to the regeneration of the articular cartilage.

## 1. Introduction

It is largely established that the regenerative power present in some regions of the body of vertebrates depends on the presence of resident stem cells localized in micro-regions known as stem cell niches [1,2,3]. The number, extension, and duration of the niches during lifetimes in different vertebrates may vary and be higher in anamniotes such as amphibians and fish that grow for most of their lifespan in comparison to birds and mammals (homethermic amniotes) that instead possess a determinate growth. In heterothermic sauropsids, tissue regeneration is generally higher than in mammals and birds, and the tail can broadly regenerate, although imperfectly, in many lizards [4,5,6,7]. It is likely that skeletally mature reptiles possess stem cell niches in body regions where growth is still active, whereas stem cell niches are reduced in mammals at skeletal maturity. Sparse stem cells niches remain in mammals, and are involved with cell replacement in organs that need a rapid cell turnover (intestine, skin, bone marrow) or are the sites of reproductive cells (gonads) [8].

One of the tissues capable of regeneration in lizards is the cartilaginous tissue of the tail vertebrae, which recovers after lesions and gives rise to a long cartilaginous tube in the regenerated tail [9,10,11,12,13,14]. In contrast, instances of cartilage regeneration are uncommon in mammals, in particular the articular cartilage of the joints such as knees or elbows [15,16].

Recent studies have illustrated the ample regenerative potential of the epiphyses of long bones in the knee of adult lizards (*Podarcis muralis*) after injury [17]. This study showed that proliferating cells are localized in the articular cartilage and in the metaphyseal growth plate of the epiphyses of the long bones, likely as a part of the continuous growth in this species. The new cartilage forms cartilaginous epiphyses which cells are mainly derived from the proliferation of chondrocytes from the articular cartilage. The study suggested that other cartilaginous cells are also derived from the methaphyseal growth centers where numerous chondroblasts are still proliferating in normal conditions. The same study postulated that proliferating cells forming the new cartilage represent a population of transient amplifying cells derived from resident stem cells localized in the epiphyses, but no direct proof has been shown.

In order to address the above hypothesis in the present study, we have analyzed the distal femur and proximal tibia bones forming the knee, after injuries. We used immunocytochemical methods to detect the presence of putative resident stem cells that replace chondrocytes in the growing centers of normal bones. This has been done using 5-Bromo-deoxyuridine and telomerase immunolabeling as an indication of stemness [18].

## 2. Results

### 2.1. General Histological Changes after Knee Injury

**Figure 1:**
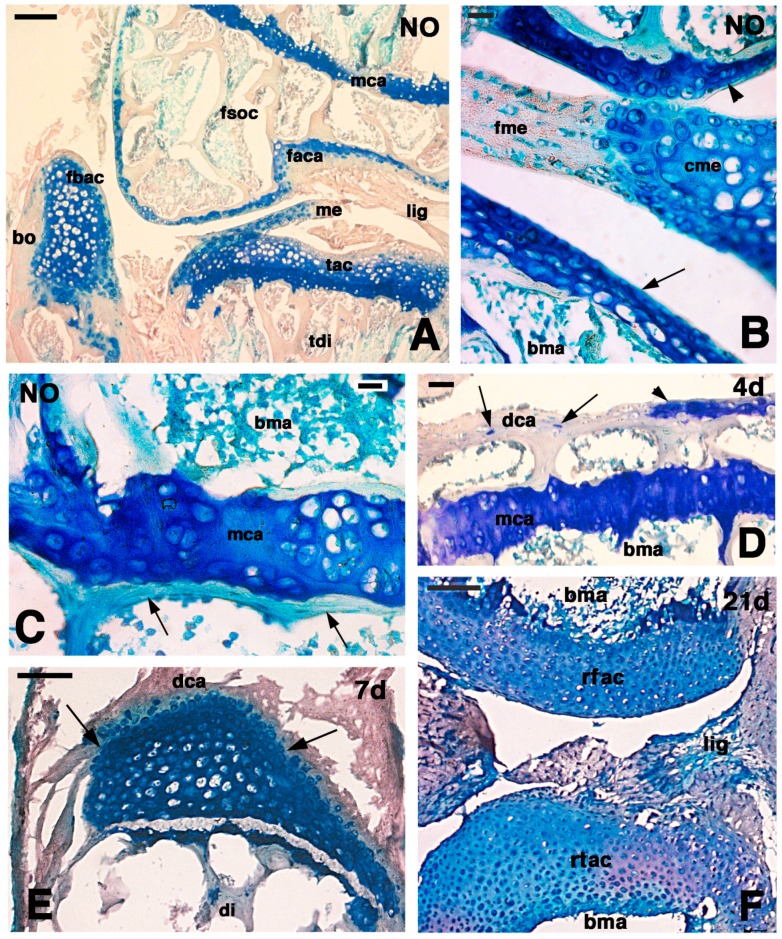
Histology of the main stages of cartilage regeneration in the knee. (**A**) detail on the epiphyses of the three long bones of the normal knee (NO) showing the extensive basophilic areas forming the articular cartilages. Bar, 50 µm; (**B**) detail on the normal articular cartilage (NO) of the femur (arrowhead) and of the tibia (arrow) with interposed the fibrous and cartilage regions of the meniscus. Bar, 10 µm; (**C**) detail of the metaphyseal growth plate bounded by a continuous bone (arrow) in the diaphyseal side of normal knee (NO). Bar, 10 µm; (**D**) loss of the basophilia in the articular cartilage of the femur four days post-injury. Only small cells (arrows) or basophilic areas (arrowhead) remain in the articular cartilage but are still extensive in the metaphyseal growth plate. Bar, 25 µm; (**E**) Nucleus of regenerating cartilage (arrows) located underneath the degenerated surface of the articular cartilage in a tibia seven days post-injury. Bar, 50 µm; (**F**) complete regenerated femur and tibia epiphyses 21 days post-injury. Note the dense cellular density of the chondrocytes. Bar, 50 µm. Legends: bo, bone; bma, bone marrow; cme, cartilage of the meniscus (lunula); di, diaphyseal bone; dca, degenerated cartilage; faca, articular cartilage of the femur; fbac, articular cartilage of the fibula; fme, fibrous connective of the meniscus; fsoc, secondary ossification center of the femur; lig, ligament; mca, metaphyseal cartilage; me, meniscus; rfac, regenerated femur articular cartilage; rtac, regenerated tibia articular cartilage; tac, articular cartilage of the tibia; tdi, tibia diaphyseal bones.

The normal knee of lizard contained cartilaginous tissue in the articular surfaces of all the three elements, femur, tibia and fibula (Figure 1A). The femur and tibia contained secondary ossification centers, seen in sections intercepting the medial axis of these bones, and located between the articular cartilage facing the synovial cavity and the metaphyseal growth plate. The secondary ossification centers were not seen in more tangential sections, where the articular cartilage appeared in continuation with the growth plate, forming an apparent thick articular cartilage (see the tibia section in Figure 1A).

The meniscus, located between the two articular cartilages of the femur and tibia, also contained a cartilaginous region or lunula (Figure 1B). The articular cartilage varied in thickness according to different areas of its surface but this tissue however contained flat chondrocytes in the superficial region and oval to rounding toward the trabecular bones of the secondary centers containing bone marrow (Figure 1B). The metaphyseal growth plate of both femur and tibia were surrounded by an almost continuous stratum of bone trabeculae in contact with hyperthrophic chondrocytes of the metaphyseal growth plate, and formed isogenous groups or even serial cartilage (piled-like, including resting and proliferating chondroblasts) (Figure 1A,C).

At four days post-lesion the superficial articular cartilage was largely degenerated, it lost its basophilia and numerous chondrocytes disappeared leaving an eosinophilic dense connective instead of a cartilaginous tissue, the contained small, probably pycnotic nuclei (Figure 1D). The metaphysis were generally not interested from the lesion, and therefore most of them conserved the typical structure and basophilia seen in the normal epiphyses. After seven days from the lesion, while the degenerating eosinophilic articular cartilage was still extensive, in some small regions of the femur and tibia epiphyses, new chondroblasts were seen and in some sections they accumulated into larger masses that gave rise to a highly cellularized cartilaginous tissue (Figure 1E). The amount of this new cartilage increased at 14 and 21 days post-lesion so that large masses of highly cellular cartilage, with relatively less intercellular ground substance were formed. The density of chondrocytes, the presence of little matrix, and of two-celled isogenous groups indicated that this cartilage was regenerated. Eventually, this process transformed the epiphyses into completely cartilaginous areas devoid of secondary centers (Figure 1F).

### 2.2. General Cell Proliferation and Cartilaginous Regeneration

**Figure 2:**
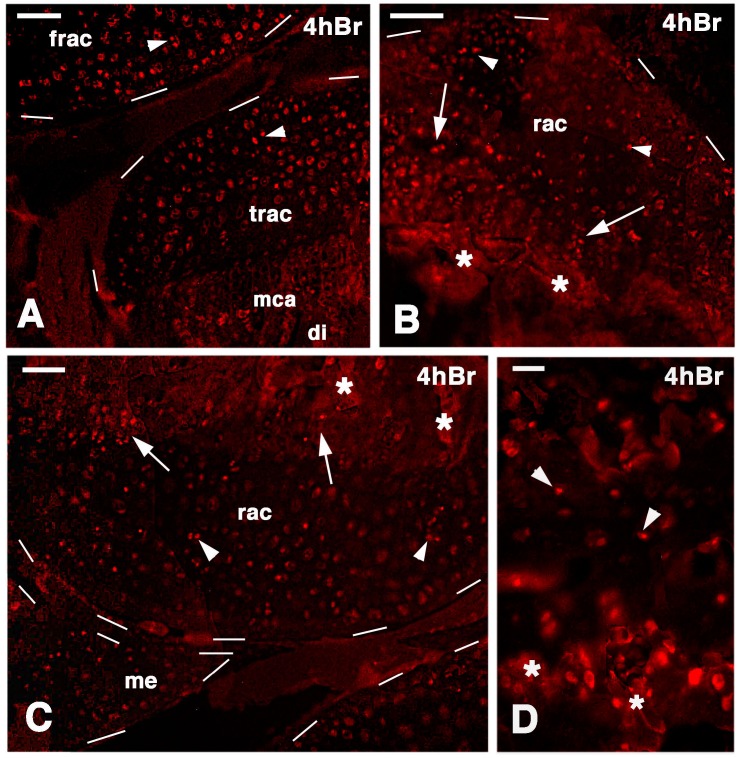
Immunofluorescence for TRITC for labeled cells in 21-day post-lesion knee epiphyses, four hours post-injection of 5Brdu. Dashes outline the cartilage surface. (**A**) sparse intensely labeled cells (arrowheads) are seen within the cartilaginous epiphyses. Bar, 50 µm; (**B**) detail on labeled cells (arrowheads) of the tibia epiphyses while labeled cells are also present in the serial cartilage (arrows) of the metaphyseal growth plate. Asterisks indicate some non-specific labeling of the mineralized matrix. Bar, 50 µm; (**C**) femur regenerated epiphyses containing inner labeled cells (arrowheads) and others labeled cells (arrows) are present in the serial cartilage of the metaphyseal growth plate and meniscus. Bar, 50 µm; (**D**) detail showing labeled cells (arrowheads) in the resting area of the forming metaphyseal growth plate. Asterisks indicate a non-specific labeling of the mineralized matrix Bar, 25 µm. Legends: frac, femur regenerated articular cartilage; me, meniscus; mca, metaphyseal articular cartilage; rac, regenerated articular cartilage; trac, tibia regenerated articular cartilage.

At 21 days post-lesion, the regenerated cartilaginous epiphyses showed numerous labeled cells at 4 h post-injection of 5BrdU. Labeled chondrocytes were seen inside the mass of the regenerating cartilage while labeled cells appeared less frequent on the synovial surface of the articular cartilage in both femur and tibia (Figure 2A–C). Sparse labeled cells were also observed in the piled chondroblasts of the metaphyseal growth plate and in the small cells of the germinal region located above the piled chondroblasts (resting and proliferative zones of the growth plate (Figure 2D). Also in the meniscus, labeled cells appeared less frequent along the surface contacting the synovial cavity while labeled cells were more commonly seen inside the meniscus tissue. Control sections were immunonegative (data not shown).

**Figure 3:**
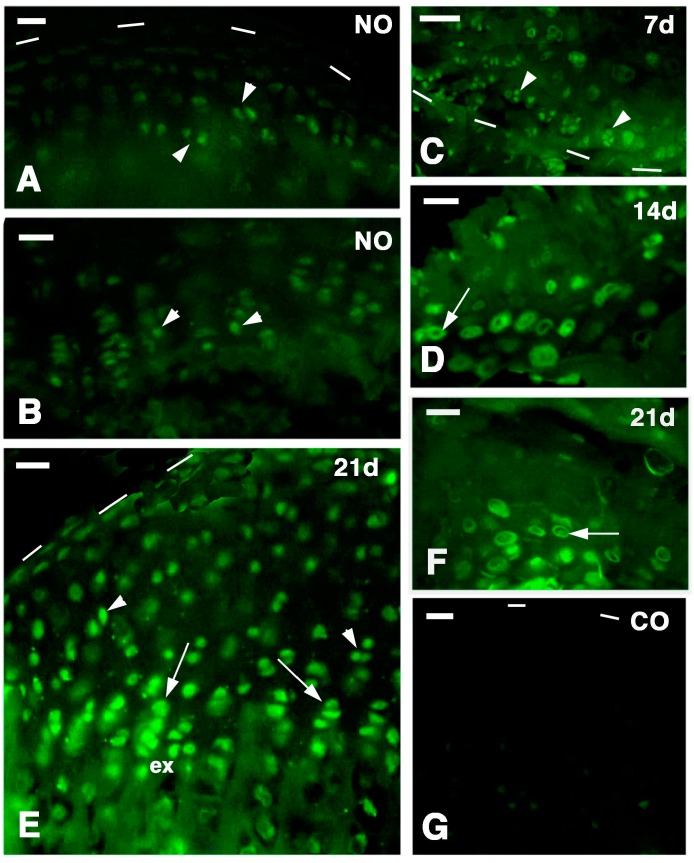
Chondroitin Sulfate Proteoglycan FITC-immunolabeling. Dashes outline the articular surface. (**A**) Articular cartilage of normal tibia (NO) showing labeling in the isogenous groups (arrowheads) but scarce in the flat chondrocytes of the surface contacting the sinovial cavity (dashes). Bar, 25 µm; (**B**) detail of the labeled cells (arrowheads) within the serial cartilage of a metaphyseal growth plate of a normal tibia (NO). Bar, 25 µm; (**C**) labeling in the chondroblasts of the isogenous groups (arrowheads) in a regenerating femur cartilage seven days after injury. Bar, 50 µm; (**D**) detail on the labeling localized in the pericellular ring-like space (arrow) of the new chondrocytes at 14 days post-lesion. Bar, 25 µm; (**E**) labeling in chondroblasts (arrowheads) of the regenerated tibia epiphyseal cartilage 21 days post-injection. The piles chondroblasts of the serial cartilage (arrows) also show a diffuse labeling in the extracellular matrix (ex). Bar, 25 µm; (**F**) Detail of the immunolabeling mainly seen in the ring-like pericellular space (arrow) around chondrocytes 21 days after injury. Bar, 25 µm; (**G**) negative control. Bar, 25 µm.

The immunostaining for a chondroitin sulfate proteoglycan, likely perlecan and aggrecan, showed that in normal epiphysis a weak immuno-reactivity was present in articular chondrocytes while the proliferating piled chondrocytes of the serial metaphyseal growth plate (resting and proliferative zone) were the most reactives (Figure 3A,B). However the strongest immuno-reaction for this chondroitin sulphated proteoglycan was observed in the cytoplasm and in the pericellular, ring-like region surrounding the regenerating chondroblasts detected at 7, 14 and 21 days of tissue regeneration (arrows in Figure 3C–F). The labeling was concentrated in the surrounding capsula (pericellular region) of roundish to hypertrophic chondrocytes located within the regenerated articular cartilage and appeared much more diffuse in the surrounding extracellular ground substance in both normal and regenerated cartilages. Also in the metaphyseal growth plate the immunofluorescence was mainly cellular and pericellular while it appeared diffuse to very low in the extracellular matrix. In control sections no immunoreactivity was seen (Figure 3G).

### 2.3. Immunodetection of Long Labeling Retaining Cells

**Figure 4:**
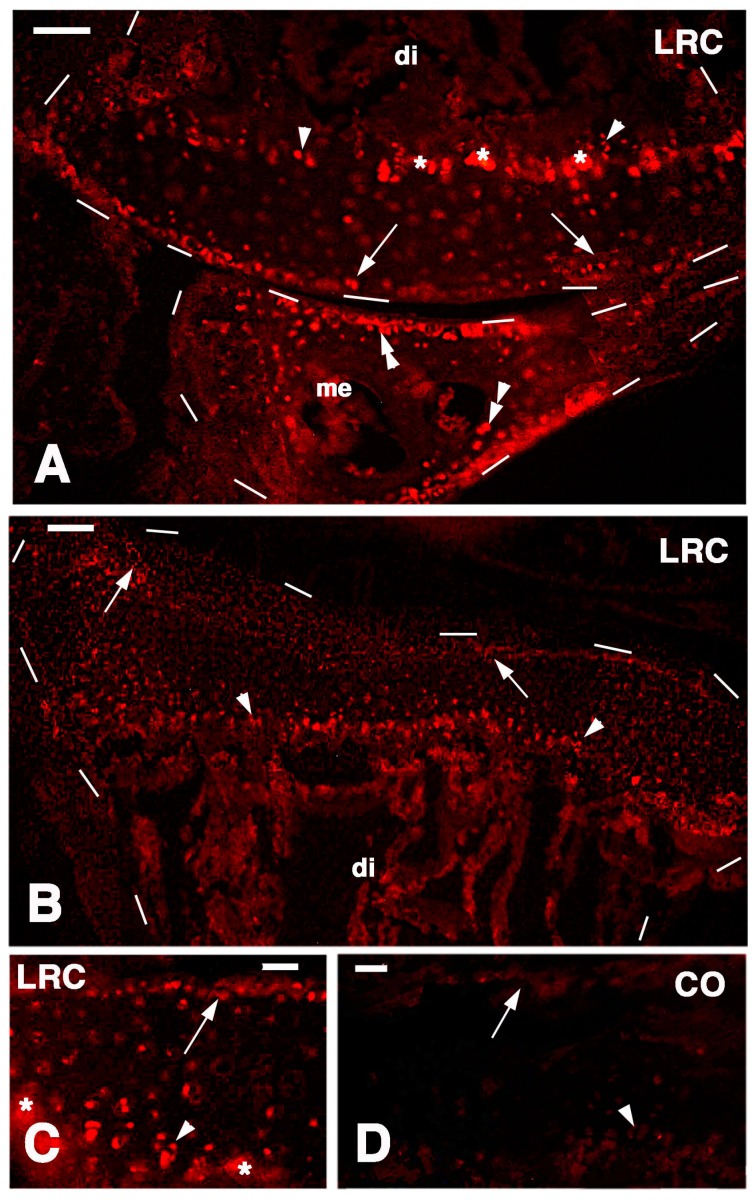
TRITC immunofluorescence for Long Retaining Cells (LRC) in normal articular surfaces of knees. (**A**) sparsely labeled cells (arrows) are seen on the surface of the femur (outlined by dashes; di, diaphysis), metaphyseal growth plate (arrowheads, asterisks indicate non-specific labeling of the mineralized matrix), and meniscus (me, double arrowheads). 50 µm; (**B**) distribution of labeled cells (arrows) in the articular surface (outlined by dashes) and metaphyseal growth plate (arrowheads). di, diaphysis. Bar, 50 µm; (**C**) detail on labeled cells of the superficial region of the articular cartilage (arrow) and serial cartilage (arrowhead) of the metaphyseal growth plate. Asterisks indicate non-specific labeling of the mineralized matrix. Bar, 25 µm; (**D**) control section with indicated the position of the articular cartilage surface (arrow) and metaphyseal growth plate (arrowhead). Bar, 25 µm.

After one week of pulse and three-and-a-half weeks of chase, some labeled nuclei of chondrocytes were still observed in normal knees, mainly sparsely along the surface of the articular cartilage of the femur and tibia. Labeled nuclei were also seen in the serial cartilage (resting and proliferating region) of the metaphyseal growth plate (arrows and arrowheads in Figure 4A,B). The labeling in the latter was a bit disturbed by the non-specific absorption of the fluorescent dye in the calcified cartilage (probably by the absorbing spongy surface formed during sectioning), but at higher magnification the nuclear labeling was evident (Figure 4C). Although less numerous, intensely labeled cells were also sparse in the lower layers of the articular cartilage and labeled cells were detected mainly along the perimeter of the meniscus and in the bone marrow. No labeling was present in control sections (Figure 4D).

**Figure 5:**
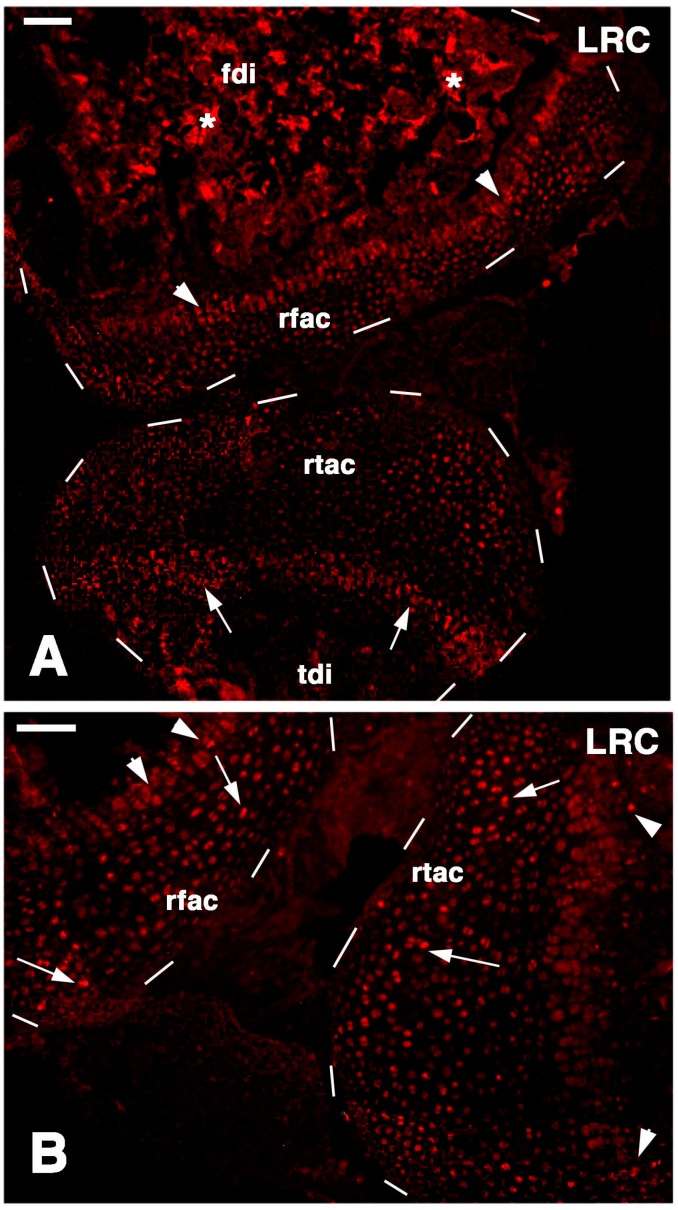
TRITC immunofluorescence for LRCs in regenerated cartilaginous epiphyses at four-and-a-half weeks post-injury. (**A**) General view of the regenerated cartilaginous articular cartilage of the femur (rfac) and tibia (rtac), both outlined by dashes. Sparse labeled cells are present within the cartilage and in the metaphyseal growth plates (arrowheads in femur, arrows in tibia). Asteriks indicate a non-specific labeling of the mineralized matrix in the femur diaphysis (fdi) and tibia diaphysis (tdi). Bar, 50 µm; (**B**) detail of the regenerated femur articular cartilage (rfac) and that of the tibia (rtac), for which surfaces are outlined by dashes. Sparse intensely labeled LRCs are seen within the cartilaginous tissue (arrows) and few also in the metaphyseal growth plate (arrowheads). Bar, 50 µm.

**Figure 6:**
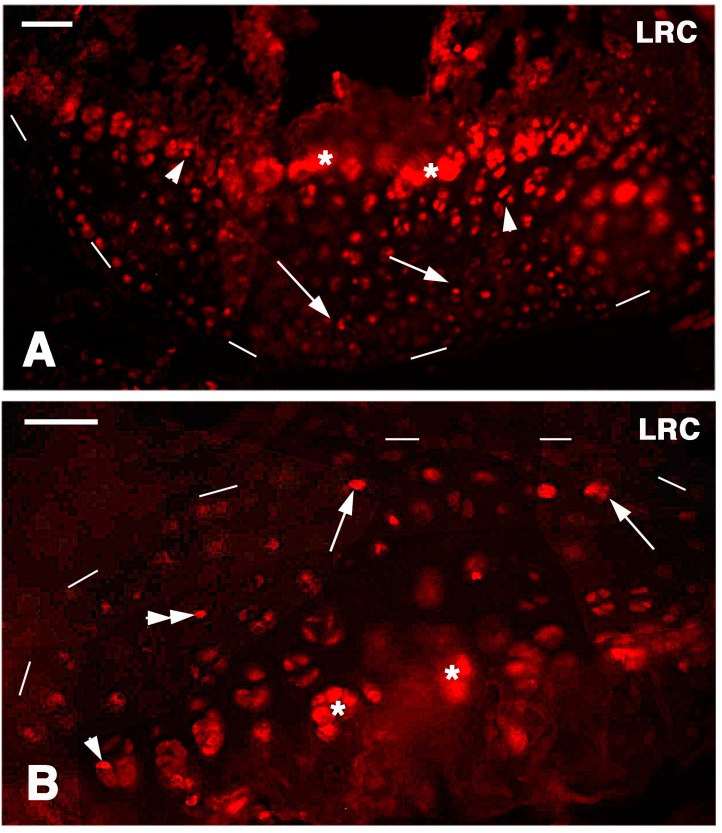
Detail on the femur (**A**) and tibia (**B**) articular surfaces (outlined by dashes) for Long Retaining Cells. (**A**) Arrows indicate some labeled cells inside the cartilage while arrowheads indicate labeled cells in the serial cartilage of the metaphyseal growth plate (asterisks indicate non-specific labeling of the mineralized matrix). Bar, 50 µm; (**B**) arrows indicate surface labeled cells, the double arrowhead point to cells within the cartilaginous tissue while the arrowhead indicates a labeled cells in the serial cartilage of the metaphyseal growth plate. Asterisks indicate non-specific labeling of the mineralized matrix around chondrocytes. Bar, 50 µm.

In the cartilaginous epiphyses present at four-and-a-half weeks post-lesion, a diffuse labeling was present but intensely labeled nuclei were only sparsely seen within all the cartilaginous tissues, and less commonly in the superficial articular cartilage while they were still present in the metaphyseal growth plate areas (Figure 5A,B and Figure 6A,B). The qualitative observations suggested that the more intensely labeled nuclei were more commonly seen in single chondroblasts irregularly sparse between the superficial articular cartilage and the metaphyseal growth plate. Occasional nuclei within isogenous groups were less intensely labeled and the label appeared weak in most of these nuclei, an indication of labeling dilution during proliferation for the regeneration of the cartilage tissue (Figure 6B). Therefore only the few sparse and more intensely labeled nuclei were considered to represent long retaining cells.

### 2.4. Telomerase Immunodetection

**Figure 7:**
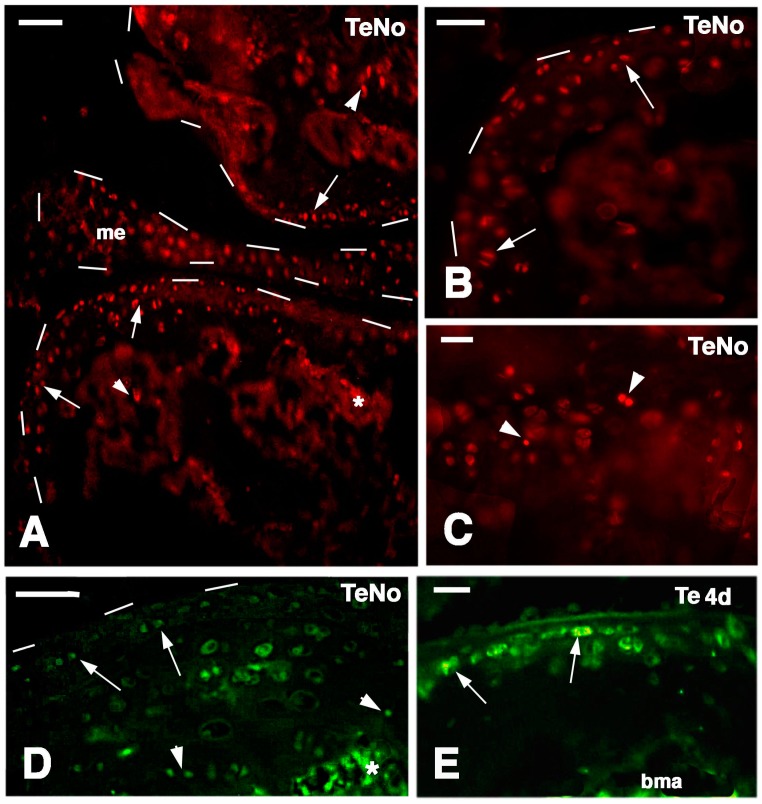
Immunofluorescence for a telomerase-1 components in normal knees (TeNo **A**–**C**, TRITC; **D**, FITC) and at four days post-injury (**E**, FITC). Dashes outline the articular cartilages and meniscus surfaces. (**A**) Sparse labeled cells are seen in the articular cartilages of the femur (top arrow) and tibia (bottom arrows), and few in the metaphyses (arrowheads). Also some labeled cells are present in the meniscus. The asterisk indicates a non-specific fluorescence of the mineralized matrix. Bar, 50 µm; (**B**) detail of labeled cells (arrows) on the surface of the articular cartilage. Bar, 50 µm; (**C**) detail on labeled cells (arrowheads) in the serial cartilage of the metaphyseal growth plate. Bar, 25 µm; (**D**) other images showing labeled cells on the articular surface (arrows) and metaphyseal growth plate (arrowheads) of a normal tibia. The asterisk indicates a non-specific fluorescence of the mineralized matrix. Bar, 50 µm; (**E**) labeled cells (arrows) in an intact area of the articular cartilage of a tibia four days after injury. bma, bone marrow. Bar, 25 µm.

The pattern of labeling in the normal knee exibited mainly small cells located along the articular cartilage surface of both femurs and tibia but also sparse labeled cells were observed along the meniscus surface, in the metaphyseal cartilage (arrows and arrowheads in Figure 7A–C) and in sparse cells of the bone marrow. The labeling was mainly nuclear although some cells also showed a cytoplasmic fluorescence (Figure 7D). In the metaphyseal (growth) plate labeled cells were sparsely seen in the small serial piled cells, not in hypertrophied chondrocytes bounding the trabecole of the diaphysis.

At four days post-lesion sparse telomerase-labeled flat cells remained on the surface of the intact articular cartilage (Figure 7E) while labeled cells disappeared in the degenerating regions.

**Figure 8:**
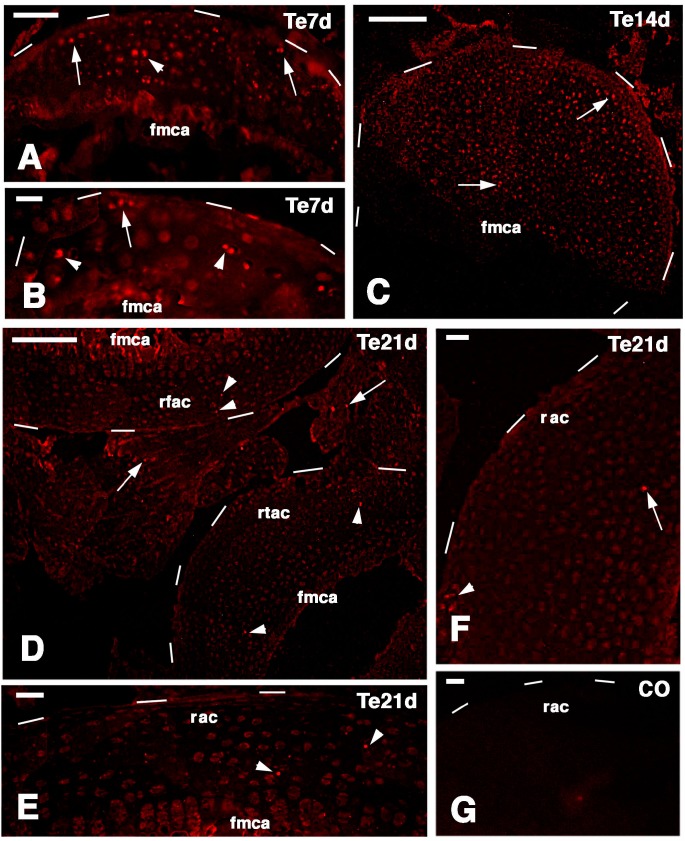
TRITC immunolabeling for a telomerase-1 components (Te) in the regenerating epiphyses (dashes outline their surfaces). (**A**) At seven days few labeled cells are seen within the new cartilage (arrowhead) and along the surface (arrows) of a tibia. Bar, 50 µm; (**B**) other mass of cartilaginous tissue in a tibia at seven days post-injury showing labeled cells on the surface (arrow) and inside the cartilaginous tissue (arrowheads). Bar, 25 µm; (**C**) sparse intensely labeled cells within (arrowhead) and near the surface (arrow) of a cartilaginous epiphyses 14 days after injury. Bar, 100 µm; (**D**) Very sparse labeled cells are seen within the femur (top arrowheads) and tibia (bottom arrowheads) articular cartilages 21 days after injury. Few labeled cells (arrows) are also seen in the meniscus. Bar, 100 µm; (**E**) detail showing two labeled cells (nuclei, arrowheads) within the regenerated cartilage at 21 days. Bar, 25 µm; (**F**) other detail on labeled cells (arrow inside the cartilage; arrowhead near the surface) at 21 days post-injury. Bar, 25 µm; (**G**) control section. Bar, 25 µm. Legends: fmca, forming metaphyseal cartilage (plate); rfac, regenerated femur articular cartilage; rac, regenerated articular cartilage; rtac, regenerated tibia articular cartilage.

In the regenerating cartilage of the epiphyses observed at seven days post-lesion, labeled cells were seen not only in the articular surface but sparse labeled cells were also present within the cartilaginous tissue in both femur and tibia (arrows and arrowheads in Figure 8A,B).

At 14 days post-lesion in the large masses of epiphyseal regenerated cartilage, the labeling was diluted but the more intensely labeled, telomerase-positive small cells were few and sparsely distributed within the entire cartilaginous mass (Figure 8C). At 21 days very sparse or occasional telomerase positive cells were seen within the regenerated cartilage of the epiphyses and in the meniscus (Figure 8D). Labeled cells were also occasionally seen on the surface of the regenerated articular cartilages and in the metaphyseal (grow) plate (Figure 8E,F). No labeling was seen in control sections (Figure 8G).

## 3. Discussion

The present study strongly suggests that the broad regeneration of the epiphyses in *P. muralis* derives from the presence of stem/primordial cells present in the articular cartilage and in the metaphyseal growth plate of the knee joint. These regions likely represent stem cell niches and correspond to the growing centers of the bones that are variably proliferating in sexually mature lizards (adults). In fact, it is likely that these reptiles continue to grow, although slowly, also after they reach sexual maturity at about two to three years of life in the species here analyzed (*P. muralis*). This appears evident from the uptake of 5BrdU in these cartilaginous areas under normal conditions, even though their epiphyses form secondary centers of ossification similar to those of mammalian epiphyses [19,20,21]. Although in some cases the normal adult knees in lizards show little cartilage and an almost complete ossification around the metaphyseal cartilage (closed metaphyses, see [15]) this definitive condition in mammalian long bones can represent only a temporary condition in other vertebrates. In fact, the apparent closure of the metaphyseal growth plate has been noted in the epiphyses of fish, amphibians and also in lepidosaurian reptiles in some periods during the year, where it is indicated as a “temporary growth cessation”, probably related with winter (resting) periods [19,20,21]. In these species the process of growth can be resumed later, possibly in spring, by the proliferation of quiescent cells (likely stem cells as indicated in the present study), and is followed by the erosion of the bone tissues around the cartilage that expands into new isogenous groups of variable extension [19].

The immunodetection of an intense immunolabeling for chondroitin Sulfate proteoglycan in regenerating chondrocytes, indicates a high cytoplasmic synthesis and secretion mainly in the ring-like, pericellular region around these cells. This observation indicates that the small chondroblasts and chondrocytes of the regenerating cartilage are actively producing the proteoglycan to be discharged in the extracellular matrix. This observation agrees with previous immunolocalization studies that showed that perlecan is especially localized in the pericellular region whereas aggregans are more widespread within the entire extracellular matrix [22]. The latter study showed that the detection of proteoglycans also in the extracellular matrix often requires treatment with chondroitinase that unmasks antigenic sites previously masked by other molecules of the matrix [22]. The lower immunoreactivity observed in chondrocytes of the normal epiphysis, further supports the hypothesis that a higher active synthesis of new matrix is present in regenerating chondrocytes, and not only in the piled serial cartilage.

**Figure 9:**
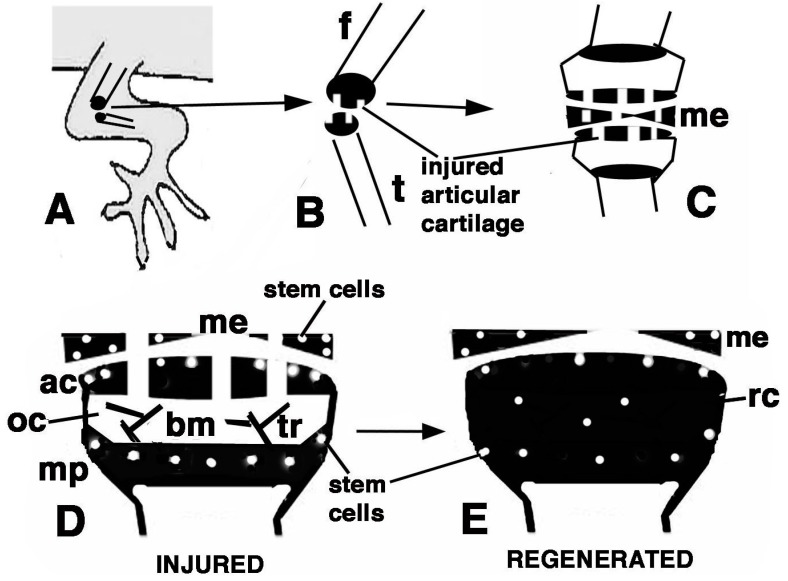
Schematic drawing resuming the present observations. (**A**) depicts a lizard knee where the injury of the articular surfaces is indicated in (**B**); (**C**) depicts the damaged area (white) produced on the articular cartilages and meniscus of the knee; (**D**) further depicts the structure of an injured epiphyses and meniscus with the position of Long Retaining Cells (LRC)/telomerase positive and putative stem cells in the articular cartilage and metaphyseal plate indicated; (**E**) depicts that the regenerated cartilage (in black) occupies the entire epiphyses at 21−32 days (four-and-a-half weeks) post-injury while stem cells appear sparse and not localized in the surface or metaphyseal plate, and are also present in the meniscus. (ac, articular cartilage; bm, bone marrow; f, femur; me, meniscus; mp, metaphyseal (growth) plate; oc, ossification center; rc, regenerated cartilage; t, tibia; tr, bone trabeculae.)

The main results of the present study on the lizard *P. muralis*, are summarized in Figure 9D,E. putative stem/progenitor cells are present on the surface of the articular cartilage and in the resting/proliferating serial chondroblasts of the metaphyseal (growth) plate. These cells likely supply new chondroblasts (transient amplifying cells) for the elongation of long bones at a high rate in the higher phase of growth taking place during the first one to three years. Unlike mammals, these cells continue to supply new chondroblasts for long bone elongation in lizards, though at a slower rate. The lizards here utilized were all adults females and males, so the main phase of growth was past, although long retaining and telomerase positive cells remained in the knees. After injury (Figure 9A–C) these stem cells are further stimulated and give rise to a surplus of transient amplifying chondroblasts that continue to proliferate and eventually form new cartilaginous epiphyses (Figure 9D,E). The present study suggests that putative stem cells are displaced in the new cartilaginous tissue within three weeks. It is unknown whether new secondary ossification centers are later re-formed and whether the remaining stem cells eventually reposition on the surface of the eroded articular cartilage. These open questions remain viable for future investigations. Previous studies conducted on mammalian sub-adults and adult articular cartilage also indicate that progenitor cells are localized in these areas [23,24,25].

Long retaining cells, some also positive for telomerase, were also observed in the bone marrow of secondary centers but their possible contribution to the regeneration of the new epiphyses remains undetermined. A previous study [18] indicated that the enzyme is particularly present in the nucleus of stem cells in the gonads, intestinal crypts and only sparsely in the blastema of the regenerating tail. Whatever the case, the presence of stem cells in the cartilage itself appears sufficient to explain the high regenerative response observed in our study as well as for the ample regeneration of cartilaginous tissues observed in other regions of the body of lizards [6,14,25,26]. The presence of stem/progenitor cells is not only limited to the cartilage but also to the cartilaginous and fibrous parts of the meniscus and also the ligaments appear to contain progenitor cells, but a further study is needed on this point. The understanding of the mechanism by which these vertebrates maintain stem cell niches in their long bones is important for studies of regenerative medicine on the articular cartilage [16,24,25,27].

## 4. Experimental Section 

### 4.1. Experiments

We used six adult individuals of Italian wall lizard (*Podarcis muralis*), including males and females longer than 12 cm. The husbandry and experimental manipulations followed the Italian guidelines for animal care and handling (Art. 5, DL 116/92). Briefly, after anesthesia using ethylic ether, the skin of the right knee of lizards was cleaned with a Lugol iodinated-solution and opened with a sharp scalpel, the muscles and connective tissues and the knee bones were exposed. The epiphyses of both femur and tibia were visually damaged under a stereomicroscope with three scissor-cuts made on the surface of the articular cartilage, taking care not to damage the femoral artery to avoid extensive bleeding (Figure 9). After the operation, the limb tissues were recomposed over the injured knee and the skin flaps reconstituted to cover the surgical incision adding healing powder (Cicatrene, Welcome Italia, Pomezia-Rome) to help maintain the skin in place over the wound.

The six operated lizards recovered well after the operation, and regained an apparent normal movement of the operated hindlimbs. They were maintained in cages with sun exposure, and at summer temperatures reaching over 30 °C during most of the day, and keeping above 20 during the night. A few hours after surgery the lizards received injection of 50 mg/kg body weight with 5-Bromo-deoxyUridine (5BrdU, Sigma, St. Louis, MO, USA) dissolved in Ringer. The injections were repeated for a week (pulse period) and later the animals were left to recover for three-and-a half weeks before sacrifice (chase period). Therefore recovery lasted four-and-a-half weeks in total from the initial injury, including the first week of pulse experiments. This group served for detecting long retaining labeled cells (putative stem cells) in the knee epiphyses and cartilage.

Two additional operated lizards, were left to recover for 3 weeks after injury, and they were injected with 5BrdU and sacrificed after 4 h as above. These specimens served for checking the general proliferation in the cartilage after 3 weeks post-injury (for both the injured and the contralateral normal knees). The other 8 lizards with injuries at progressive periods were utilized for histology and for the immunodetection of telomerase. From these animals, the injured knees were collected and fixed as above at 4 days post-injury (*n* = 2), 7 day post-injury (*n* = 2), 14 days post-injury (*n* = 2), and 21 days post-injury (*n* = 2).

### 4.2. Tissue Preparation and Microscopic Techniques

The operated and the contra-lateral normal knees were collected at about half of the length of both femur and tibia, and immediately immersed in 4% paraformaldehyde at 0–4 °C for about 4 h. During this time non-bone tissue around the knee bones were removed in order to allow a better and more rapid penetration of the fixative. The samples were immersed for 18–20 h (with two changes) in a post-fixative decalcifying solution (5% formic acid, 15% formaldehyde at 35% concentration, and 80% distilled water in volumes) at room temperature. Later, the tissues were dehydrated, clarified in xylene, and embedded in wax for the following histological and immunocytochemical study.

Sections along the longitudinal plane were obtained using a microtome (Reichert, Depew, NY, USA) at 6–9 μm in thickness, and collected on glass slide pre-coated with albumin-chromoalum. After de-waxing and dehydration, some sections were stained with 1% methylene blue and 1% Eosin, for the histological study. Other sections containing the femur and tibia, and in some cases also the fibula, were instead utilized for the immunocytochemical detection.

Sections were pre-incubated for 30 min with a 0.1 M Tris buffer solution at pH 7.6 containing 2% Bovine Serum Albumin (Sigma, St. Louis, MO, USA) and 5% Normal Goat Serum (Sigma), to saturate un-specific binding sites. Part of the sections were incubated with a mouse anti-5BrdU antibody at 1:100 dilution in the buffer for 5–6 h at room temperature and then rinsed in the buffer (in control sections the primary antibody was omitted from the solution). The 5BrdU-monoclonal antiserum (mouse G3G4) was developed by Dr. S.J. Kaufmann of the University of Illinois at Urbana, USA, and was provided by the Developmental Studies Hybridoma Bank, maintained by the University of Iowa, Iowa City, USA. This immunodetection aimed to detect long retaining labeled cells, indicated as putative stem cells.

A second mouse antibody against a chondroitin sulfate proteoglycan (MAB2029, Chemicon Int., Tamecula, CA, USA) was utilized at 1:150 dilution in buffer in order to detect chondroblasts in active phase of secretion, including possible regenerating chondroblasts. Other sections were instead stained for a rabbit antibody produced against a lizard telomerase-1 component. The latter antibody has labeled germinal and intestinal stem cells, and is indicative for stemness [18]. The sections were later incubated with a goat anti-mouse or anti-rabbit TRITC-conjugated secondary antibody (Tetramethyl Rhodamine Isothiocyanate, Sigma) or FITC (Fluoresceine Isothiocyanate, Sigma) diluted 1:100 in the above buffer solution. The immunoreacted sections were observed and photographed using an Epifluorescence microscope (Euromex, Arnhem, The Netherlands) equipped with a Rhodamine (Euromex) or Fluorescein filter (Euromex).

## 5. Conclusions

In conclusion, this study shows that the broad regenerative power of epiphyseal cartilages in lizards depends on the permanence of 5BrdU long labeling retaining cells as well as some also showing presence of telomerase, representing putative stem cells niches localized in their growing centers. It appears that this condition is linked to the slow but potentially continuous growth throughout most of the life in these sauropsids. Since lizards are amniotes relatively closer to the mammalian condition than amphibians, the understanding of the factors that allow the persistence of chondrogenic stem/progenitor cell niches at the extremities of their long bones should shed light on the mechanisms for the establishment of stem cell niches in vertebrates.
